# Multimorbidity, cognitive phenotypes, and Alzheimer's disease plasma biomarkers in older adults: A population‐based study

**DOI:** 10.1002/alz.13519

**Published:** 2023-12-02

**Authors:** Yifei Ren, Yuanjing Li, Na Tian, Rui Liu, Yi Dong, Tingting Hou, Cuicui Liu, Xiaolei Han, Xiaodong Han, Lidan Wang, Davide Liborio Vetrano, Tiia Ngandu, Alessandra Marengoni, Miia Kivipelto, Yongxiang Wang, Lin Cong, Yifeng Du, Chengxuan Qiu

**Affiliations:** ^1^ Department of Neurology Shandong Provincial Hospital Shandong University Jinan Shandong P. R. China; ^2^ Aging Research Center and Center for Alzheimer Research Department of Neurobiology Care Sciences and Society Karolinska Institutet‐Stockholm University Solna Sweden; ^3^ Department of Neurology Shandong Provincial Hospital affiliated to Shandong First Medical University Jinan Shandong P. R. China; ^4^ Shandong Provincial Clinical Research Center for Geriatric Neurological Diseases Jinan Shandong P. R. China; ^5^ Medical Science and Technology Innovation Center Shandong First Medical University & Shandong Academy of Medical Sciences Jinan Shandong P. R. China; ^6^ Stockholm Gerontology Research Center Stockholm Sweden; ^7^ Department of Public Health and Welfare Finnish Institute for Health and Welfare Helsinki Finland; ^8^ Division of Clinical Geriatrics Center for Alzheimer Research Department of Neurobiology Care Sciences and Society Karolinska Institutet Solna Sweden; ^9^ Department of Clinical and Experimental Sciences University of Brescia Brescia Italy; ^10^ Theme Inflammation and Aging Medical Unit Aging Karolinska University Hospital Stockholm Sweden; ^11^ Institute of Public Health and Clinical Nutrition University of Eastern Finland Joensuu Finland; ^12^ The Ageing Epidemiology Research Unit School of Public Health Imperial College London London UK; ^13^ Institute of Brain Science and Brain‐Inspired Research Shandong First Medical University & Shandong Academy of Medical Sciences Jinan Shandong China

**Keywords:** dementia, mild cognitive impairment, multimorbidity, plasma biomarkers, population‐based study, rural

## Abstract

**INTRODUCTION:**

To examine the burden and clusters of multimorbidity in association with mild cognitive impairment (MCI), dementia, and Alzheimer's disease (AD)‐related plasma biomarkers among older adults.

**METHODS:**

This population‐based study included 5432 participants (age ≥60 years); of these, plasma amyloid beta (Aβ), total tau, and neurofilament light chain (NfL) were measured in a subsample (*n* = 1412). We used hierarchical clustering to generate five multimorbidity clusters from 23 chronic diseases. We diagnosed dementia and MCI following international criteria. Data were analyzed using logistic and linear regression models.

**RESULTS:**

The number of chronic diseases was associated with dementia (multivariable‐adjusted odds ratio = 1.22; 95% confidence interval [CI] = 1.11 to 1.33), AD (1.13; 1.01 to 1.26), vascular dementia (VaD) (1.44; 1.25 to 1.64), and non‐amnestic MCI (1.25; 1.13 to 1.37). Metabolic cluster was associated with VaD and non‐amnestic MCI, whereas degenerative ocular cluster was associated with AD (*p* < 0.05). The number of chronic diseases was associated with increased plasma Aβ and NfL (*p* < 0.05).

**DISCUSSION:**

Multimorbidity burden and clusters are differentially associated with subtypes of dementia and MCI and AD‐related plasma biomarkers in older adults.

**Highlights:**

- We used hierarchical clustering to generate five clusters of multimorbidity.
- The presence and load of multimorbidity were associated with dementia and mild cognitive impairment.
- Multimorbidity clusters were differentially associated with subtypes of dementia and Alzheimer's disease plasma biomarkers.

## 1 BACKGROUND

Multimorbidity, usually defined as the coexistence of two or more chronic health conditions, contributes to global health inequalities of an aging population.^1^ In the past decade, several population‐based studies have shown that multimorbidity is associated with accelerated cognitive decline, mild cognitive impairment (MCI), and dementia.^2^, ^3^, ^4^, ^5^, ^6^, ^7^, ^8^ However, most of these studies have been conducted among urban populations of high‐income countries in Europe and North America,^4^, ^5^, ^9^ which limited the generalizability of research findings to other populations, especially the rural populations of low‐ and middle‐income countries. This is relevant given that epidemiological features (eg, prevalence, distribution, and risk factors) of chronic conditions and dementia disorders differ considerably between high‐income and low‐ and middle‐income countries as well as between urban and rural populations.^10^, ^11^, ^12^, ^13^

Some chronic conditions tend to occur together in older adults due to shared risk factors or common pathophysiological mechanisms,^14^, ^15^ so it is important to define different clusters of multimorbidity when investigating multimorbidity.^16^ Indeed, previous studies showed that the associations of multimorbidity with dementia varied by different patterns of multimorbidity,^4^ suggesting the importance of assessing specific disease clusters when studying the relationship of multimorbidity with cognitive phenotypes. Furthermore, the relationships of the load and clusters of multimorbidity with Alzheimer's disease (AD)‐related plasma biomarkers have yet to be explored in the general population settings. This is crucial to understanding the potential mechanisms underlying the associations of multimorbidity burden and clusters with cognitive phenotypes (eg, MCI and dementia).

Therefore, in this population‐based study of rural‐dwelling older adults in China, we aimed to investigate the association of multimorbidity with cognitive aging phenotypes and plasma AD‐related biomarkers. We hypothesized that the greater burden and certain clusters (eg, metabolic cluster) of multimorbidity would be associated with a higher likelihood of MCI and dementia, as well as with plasma AD‐related biomarkers.

## 2 METHODS

### 2.1 Study populations

**FIGURE 1 alz13519-fig-0001:**
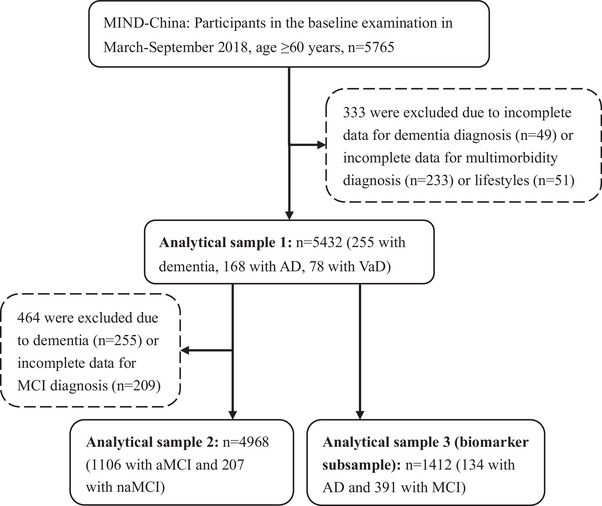
Flowchart of study participants. AD, Alzheimer's disease; aMCI, amnestic mild cognitive impairment; MCI, mild cognitive impairment; MIND‐China, Multimodal Interventions to Delay Dementia and Disability in Rural China; naMCI, non‐amnestic mild cognitive impairment; VaD, vascular dementia.

This population‐based study used data from the Multimodal Interventions to Delay Dementia and Disability in Rural China (MIND‐China) project, as previously described in detail.^17^, ^18^ In brief, MIND‐China was part of the World‐Wide FINGERS Network.^19^ The baseline assessment of MIND‐China targeted people aged ≥60 years by the end of 2017 and living in rural communities (52 villages) of the town of Yanlou, Yanggu County, Western Shandong Province, China. In March to September 2018, a total of 5765 participants (74.9% of all eligible persons) underwent a baseline examination. Of those, we excluded 49 subjects who had incomplete data on dementia diagnosis and an additional 284 subjects who had missing data on chronic conditions or lifestyles. Thus, the analytical sample for examining the association between multimorbidity and dementia included 5432 subjects. Of these, we further excluded 255 subjects with prevalent dementia and 209 subjects with missing diagnosis of MCI, leaving 4968 subjects for the analysis concerning the association between multimorbidity and MCI. Of these 5432 subjects, data on plasma AD‐related biomarkers were available in 1412 persons, including 134 with AD and 391 with MCI. This biomarker sample was selected using the cluster (village)‐based sampling approach that consisted of 1278 participants from 18 villages that were randomly selected from all 52 villages plus 134 persons with AD identified from other villages. People with plasma biomarkers (*n* = 1412) were slightly younger (mean age 70.15 vs 70.91 years, *p* < 0.001) and more likely to be women (61.26% vs 56.12%, *p* < 0.001) than those who did not have data on the plasma biomarkers (*n* = 4020). Figure 1 shows a flowchart of the study participants.

The MIND‐China protocol was approved by the ethics committee at Shandong Provincial Hospital Affiliated to Shandong First Medical University in Jinan, Shandong, China. Written informed consent was obtained from all participants or, in the case of cognitively impaired persons, from a proxy (usually a guardian or a family member). The MIND‐China study was registered in the Chinese Clinical Trial Registry (registration no.: ChiCTR1800017758).

### 2.2 Data collection

In March to September 2018, trained medical staff collected data following a structured questionnaire through face‐to‐face interviews, clinical and neurological examinations, neuropsychological testing, and laboratory tests, as previously reported.^17^, ^18^ The questionnaire included information on sociodemographic factors (age, sex, education, and occupation), lifestyle choices (smoking, alcohol drinking, and physical inactivity), health conditions (eg, hypertension, diabetes, coronary heart disease, and stroke), and use of medications (eg, antihypertensive agents, glucose‐lowering drugs, and lipid‐lowering agents). All medications were classified and coded according to the Anatomical Therapeutic Chemical Classification System.^20^ Arterial blood pressure was measured on the right upper arm in the sitting position after at least a 5‐min rest using an electronic blood pressure monitor (Omron HEM‐7127J; Omron Corporation, Kyoto, Japan). After an overnight fast, peripheral blood samples were taken, and fasting blood glucose and lipids were measured at the clinical laboratory of Yanlou Hospital.^17^

**RESEARCH IN CONTEXT**

1. **Systematic review**: We searched PubMed for literature. Emerging evidence has linked multimorbidity with dementia, but the associations of multimorbidity with dementia and mild cognitive impairment (MCI) have been rarely investigated in Asian populations. Furthermore, the relationship between multimorbidity clusters and Alzheimer's plasma biomarkers has yet to be explored.
2. **Interpretation**: This population‐based cross‐sectional study of rural Chinese older adults showed that multimorbidity, especially metabolic and cardiac‐musculoskeletal clusters, were associated with dementia, vascular dementia (VaD), and non‐amnestic MCI (naMCI), as well as plasma Aβ42 and NfL. In addition, degenerative ocular cluster was associated with Alzheimer's disease (AD) and plasma NfL. These results suggest that amyloid and neurodegenerative pathologies may underline the associations of multimorbidity load and clusters with dementia.
3. **Future directions**: Future prospective cohort studies should investigate the longitudinal associations of multimorbidity load and clusters with dementia and subtypes of dementia as well as the neuropathological mechanisms underlying the associations.

### 2.3 Assessments of chronic conditions and multimorbidity

In total, 23 chronic conditions were defined based on clinical examination, instrumental examination (eg, electrocardiogram examination and abdominal ultrasound examination), self‐reported health history, use of medications, and laboratory tests.^21^, ^22^ Hypertension was defined as systolic pressure ≥140 mmHg, diastolic pressure ≥90 mmHg, or current use of antihypertensive drugs. Diabetes was defined according to self‐reported physician diagnosis of diabetes, fasting blood glucose ≥7.0 mmol/L, or current use of antidiabetic agents. Hyperlipidemia was defined as total serum cholesterol ≥6.22 mmol/L, triglyceride ≥2.26 mmol/L, low‐density lipoprotein cholesterol ≥4.14 mmol/L, high‐density lipoprotein cholesterol < 1.04 mmol/L, or current use of lipid‐lowering agents.^23^ Ischemic heart disease was identified according to self‐reported history of myocardial infarction, angina pectoris, coronary intervention, or pathological Q waves on electrocardiogram. Atrial fibrillation was identified according to self‐reported history of atrial fibrillation or evidence from electrocardiogram. History of clinical stroke was ascertained according to self‐reported history of stroke or neurological examination by a neurologist. Epilepsy was identified according to self‐reported epilepsy history or current use of antiepileptic drugs. Asthma was identified according to self‐reported asthma history or current use of anti‐asthmatic drugs. Chronic kidney disease was ascertained according to self‐reported history of chronic kidney disease or estimated glomerular filtration rate ≤60 mL/min/1.73 m^2^. Thyroid disease includes hypothyroidism and hyperthyroidism, which were ascertained according to self‐reported history of the diseases. Peptic ulcer (gastric ulcer and duodenal ulcer) was defined according to self‐reported medical history or currently using proton pump inhibitor or H2 receptor antagonist. Degenerative disc disease (ie, cervical and lumbar spondylopathy), gall bladder disease (cholecystitis and cholelithiasis), chronic obstructive pulmonary disease, Parkinson's disease, heart failure, cancer, arthritis, tuberculosis, hepatitis, cataract, glaucoma, and lower extremity varicose veins were identified according to self‐reported medical history. Multimorbidity was defined as the concurrent presence in the same individual of two or more of the aforementioned 23 chronic health conditions.^24^, [Fig alz13519-fig-0001]

### 2.4 Measurement of plasma biomarkers

After an overnight fast, peripheral blood samples were drawn into ethylene diamine tetraacetic acid (EDTA) citrate vacutainer tubes and centrifuged in a tabletop centrifuge. Plasma samples were then aliquoted and stored at −80°C until being retrieved and thawed on ice. Plasma biomarkers were measured using the single‐molecule array (Simoa) platform (Quanterix Corp, MA, USA) for Aβ42, Aβ40, and total tau (Human Neurology 3‐Plex A assay), and NfL (NF‐light advantage kit) at the laboratory of Wayen Biotechnologies Inc., Shanghai, China. Two quality control plasma samples were run in duplicate on each plate for each analyte. The intra‐assay coefficient of variation and the interassay coefficient of variation were all below 13.0% for the control sample.

### 2.5 Neuropsychological assessments

A neuropsychological test battery was used to assess cognitive function, as described previously.^17^, ^18^ In brief, subjective cognitive decline was assessed via three questions of memory problems that the participants experienced in the past year, that is, difficulty remembering, forgetting what had been planned, and worry about memory decline. We used the Chinese version of the Mini‐Mental State Examination (MMSE) to assess global cognitive function and the Chinese version of activities of daily living (C‐ADLs) to evaluate self‐care and instrumental ADLs. We assessed function of four specific cognitive domains: episodic memory (Auditory Verbal Learning Test‐immediate recall, long‐delayed free recall, and long‐delayed recognition), verbal fluency (Verbal Fluency Test‐categories of animals, fruits, and vegetables), attention (Digit Span Test‐forward and Trail Making Test Part A), and executive function (Digit Span Test‐backward and Trail Making Test Part B).^25^, ^26^

### 2.6 Diagnosis of dementia and mild cognitive impairment

The diagnostic procedures and criteria of dementia and MCI were described in previous studies.^17^, ^18^ Briefly, dementia was clinically diagnosed according to the *Diagnostic and Statistical Manual of Mental Disorders*, Fourth Edition (DSM‐IV), criteria,^27^ in which a three‐step diagnostic procedure was followed,^18^ that is, the trained clinicians and interviewers conducted the first in‐person interviews, routine clinical examination, and testing to collect data on medical history, cognitive function, and C‐ADLs and recorded all the information following the structured questionnaires. Then neurologists specialized in dementia diagnosis and care reviewed all the records documented in the first step and made a preliminary diagnosis for participants who were suspected to have dementia. Finally, the neurologists conducted further face‐to‐face interviews with persons who were suspected to have dementia or who had insufficient data for making a diagnosis of dementia status or informants and reassessed their medical history, cognitive status, C‐ADLs, and, whenever available, neuroimaging data. In the case of uncertainty, a senior neurologist was consulted, and a consensus diagnosis of dementia was reached. We further classified dementia into AD according to the National Institute on Aging‐Alzheimer's Association criteria for probable AD dementia^28^ and classified vascular dementia (VaD) following the National Institute of Neurological Disorders and Stroke and the Association Internationale pour la Recherche et l'Enseignement en Neurosciences criteria for probable VaD.^29^ Dementia cases who could not be classified as AD or VaD were considered to have other types of dementia.

We defined MCI and subtypes of MCI following the Petersen criteria,^30^ to which an operational approach that integrated neuropsychological test with clinical consensus was applied, as previously described.^17^ In brief, the Petersen criteria for defining MCI included (1) cognitive concern by the subject (according to responses to the three questions of memory problems), informant or physician (Clinical Dementia Rating ≥ 0.5); (2) objective cognitive impairment evidenced in at least one of the four cognitive domains (cognitive *z*‐score ≥1.0 standard deviation (SD) below mean scores of the age‐ and education‐specific groups); (3) essentially preserved function of daily activities; and (4) absence of dementia.^17^ We categorized MCI into amnestic MCI (aMCI) if the memory domain was impaired or otherwise non‐aMCI (naMCI).

### 2.7 Statistical analysis

We used the hierarchical clustering method based on the VARCLUS procedure in SAS version 9.4 software (SAS Institute Inc., 2013, Cary, NC, USA) to generate clusters of multimorbidity.^31^ We used binary and multinomial logistic regression models to examine the associations of number of chronic conditions, multimorbidity, and different multimorbidity clusters with dementia, MCI, and their subtypes. Data on plasma biomarkers were transformed according to the method, as described in the previous study.^32^ Specifically, plasma biomarkers that were not normally distributed were first log‐transformed using the natural logarithm to minimize the impact of outliers. Then all the plasma biomarkers were standardized into *z*‐scores to enable comparison of the effect size among different biomarkers. The general linear regression models were used to examine the associations of multimorbidity measures with plasma AD‐related biomarkers. The analyses were adjusted for age, sex, education, current smoking, alcohol consumption, and physical inactivity. SAS version 9.4 was used for all statistical analyses. Two‐tailed *p* < 0.05 was considered statistically significant, and Bonferroni correction was performed to adjust for multiple comparisons.

## 3 RESULTS

### 3.1 Characteristics of study participants

The mean age of the 5432 participants was 70.71 years (SD, 5.73), 57.46% were women, 39.73% had no formal schooling, and 83.32% were farmers (Table 1). Compared to men, women were less educated and more likely to be farmers and to have hypertension, diabetes, hyperlipidemia, ischemic heart disease, thyroid disease, cataract, gallbladder disease, and degenerative disc disease and less likely to have chronic obstructive pulmonary disease, asthma, and lower extremity varicose veins (Table 1). The overall prevalence of concurrently having 1, 2, 3, 4, and ≥5 chronic health conditions was 29.12%, 28.22%, 18.94%, 8.27%, and 4.60%, respectively, in the total sample (Figure S1).

**TABLE 1:** Characteristics of study participants in total sample and by sex.

	Total sample (*n* = 5432)	Sex		
Characteristics		Male (*n* = 2311)	Female (*n* = 3121)	*P* value
Age (years), mean (SD)	70.71 (5.73)	70.60 (5.58)	70.80 (5.84)	0.209
Educational level, *n* (%)				<0.001
No formal schooling	2158 (39.73)	295 (12.77)	1863 (59.69)	
Primary school	2315 (42.62)	1219 (52.75)	1096 (35.12)	
Middle school or above	959 (17.65)	797 (34.49)	162 (5.19)	
Farmers, *n* (%)	4506 (83.32)	1571 (68.25)	2935 (94.49)	<0.001
Current smoking, *n* (%)	1142 (21.02)	1104 (47.77)	38 (1.22)	<0.001
Alcohol consumption, *n* (%)	1573 (28.96)	1365 (59.07)	208 (6.66)	<0.001
Physical inactivity, *n* (%)	1909 (35.14)	752 (32.54)	1157 (37.07)	<0.001
Multimorbidity, *n* (%)	3261 (60.03)	1270 (54.95)	1991 (63.79)	<0.001
Multimorbidity clusters, *n* (%)				
Metabolic cluster	3089 (56.87)	1191 (51.54)	1898 (60.81)	<0.001
Cardiac‐MSK cluster	1881 (34.63)	682 (29.51)	1199 (38.42)	<0.001
Degenerative ocular cluster	286 (5.27)	95 (4.11)	191 (6.12)	<0.001
Respiratory cluster	409 (7.53)	237 (10.26)	172 (5.51)	<0.001
Mixed cluster	476 (8.76)	214 (9.26)	262 (8.39)	<0.001
Chronic conditions, *n* (%)				
Hypertension	3638 (66.97)	1496 (64.73)	2142 (68.63)	0.003
Diabetes mellitus	787 (14.49)	268 (11.60)	519 (16.63)	<0.001
Hyperlipidemia	1321 (24.32)	374 (16.18)	947 (30.34)	<0.001
Ischemic heart disease	1168 (21.50)	404 (17.48)	764 (24.48)	<0.001
Heart failure	149 (2.74)	59 (2.55)	90 (2.88)	0.461
Atrial fibrillation	81 (1.49)	38 (1.64)	43 (1.38)	0.423
Stroke	863 (15.89)	388 (16.79)	475 (15.22)	0.118
Chronic obstructive pulmonary disease	405 (7.46)	252 (10.90)	153 (4.90)	<0.001
Asthma	120 (2.21)	70 (3.03)	50 (1.60)	<0.001
Epilepsy	9 (0.17)	5 (0.22)	4 (0.13)	0.429
Parkinson's disease	39 (0.72)	22 (0.95)	17 (0.54)	0.079
Cancer	72 (1.33)	31 (1.34)	41 (1.31)	0.930
Peptic ulcer	246 (4.53)	120 (5.19)	126 (4.04)	0.043
Thyroid disease	74 (1.36)	17 (0.74)	57 (1.83)	<0.001
Arthritis	927 (17.07)	373 (16.14)	554 (17.75)	0.119
Tuberculosis	93 (1.71)	48 (2.08)	45 (1.44)	0.074
Hepatitis	54 (0.99)	22 (0.95)	32 (1.03)	0.788
Chronic kidney disease	131 (2.41)	46 (1.99)	85 (2.72)	0.082
Cataract	292 (5.38)	94 (4.07)	198 (6.34)	<0.001
Gall bladder disease	76 (1.40)	21 (0.91)	55 (1.76)	0.008
Glaucoma	34 (0.63)	11 (0.48)	23 (0.74)	0.228
Lower extremity varicose veins	65 (1.20)	37 (1.60)	28 (0.90)	0.018
Degeneration disc disease	245 (4.51)	84 (3.63)	161 (5.16)	0.008

### 3.2 Associations of multimorbidity with dementia and mild cognitive impairment

Of the 5432 participants, 3261 (60.03%) were determined to have multimorbidity (≥2 chronic health conditions). In addition, 255 were diagnosed with dementia (168 with AD, 78 with VaD, and nine with other types of dementia) and 1313 with MCI (1106 with aMCI and 207 with naMCI). As a continuous variable, the number of chronic conditions was significantly associated with a ∼22% increased likelihood for dementia, ∼13% for AD, and ∼44% for VaD (Table 2). In addition, having multimorbidity was significantly associated with a 1.45‐fold increased likelihood of dementia and a 2.75‐fold increased likelihood of VaD, but not with AD (Table 2).

**TABLE 2:** Associations of multimorbidity and multimorbidity clusters with dementia, Alzheimer's disease, and vascular dementia (*n* = 5432).

			Dementia		Alzheimer's disease		Vascular dementia	
Multimorbidity burden and clusters	No. of subjects	No. of non‐demented subjects	No. of cases	Odds ratio (95% CI)^a^	No. of cases	Odds ratio (95% CI)^a^	No. of cases	Odds ratio (95% CI)^a^
No. of chronic diseases	5432	5177	255	1.22 (1.11–1.33)^*^, ^†^	168	1.13 (1.01–1.26)^*^, ^‡^	78	1.44 (1.25–1.64)^*^, ^†^
Multimorbidity								
No	2171	2094	77	1.00 (reference)	57	1.00 (reference)	15	1.00 (reference)
Yes	3261	3083	178	1.45 (1.09–1.93)^*^, ^†^	111	1.20 (0.85–1.68)	63	2.75 (1.55–4.88)^*^, ^†^
Multimorbidity clusters^b^								
Metabolic cluster	3089	2918	171	1.47 (1.11–1.96)^*^, ^†^	106	1.20 (0.85–1.69)	61	2.83 (1.59–5.03)^*^, ^†^
Cardiac‐MSK cluster	1881	1783	98	1.35 (0.98–1.85)	69	1.25 (0.86–1.82)	27	2.01 (1.05–3.85)^*^, ^‡^
Degenerative ocular cluster	286	264	22	1.70 (1.01–2.86)^*^, ^‡^	18	1.81 (1.01–3.25)^*^, ^‡^	4	1.80 (0.58–5.60)
Respiratory cluster	409	387	22	1.22 (0.72–2.05)	16	1.28 (0.70–2.36)	5	1.23 (0.43–3.54)
Mixed cluster	476	456	20	1.17 (0.69–1.97)	16	1.31 (0.72–2.39)	4	1.12 (0.37–3.40)

Abbreviations: CI, confidence interval; MSK, musculoskeletal conditions.

^a^ Odds ratio and 95% confidence interval were derived from binary and multinomial logistic regression models that were adjusted for age, sex, education, current smoking, alcohol consumption, and physical inactivity.

^b^ Participants without multimorbidity (n = 2171) were considered the reference group in estimating odds ratios (95% confidence intervals) of dementia and subtypes of dementia associated with various clusters of multimorbidity.

* *p* ≤ 0.05 in uncorrected tests.

^†^ *p* ≤ 0.05 in Bonferroni correction tests for multiple comparisons.

^‡^ *p* > 0.05 in Bonferroni correction tests for multiple comparisons.

**FIGURE 2:**
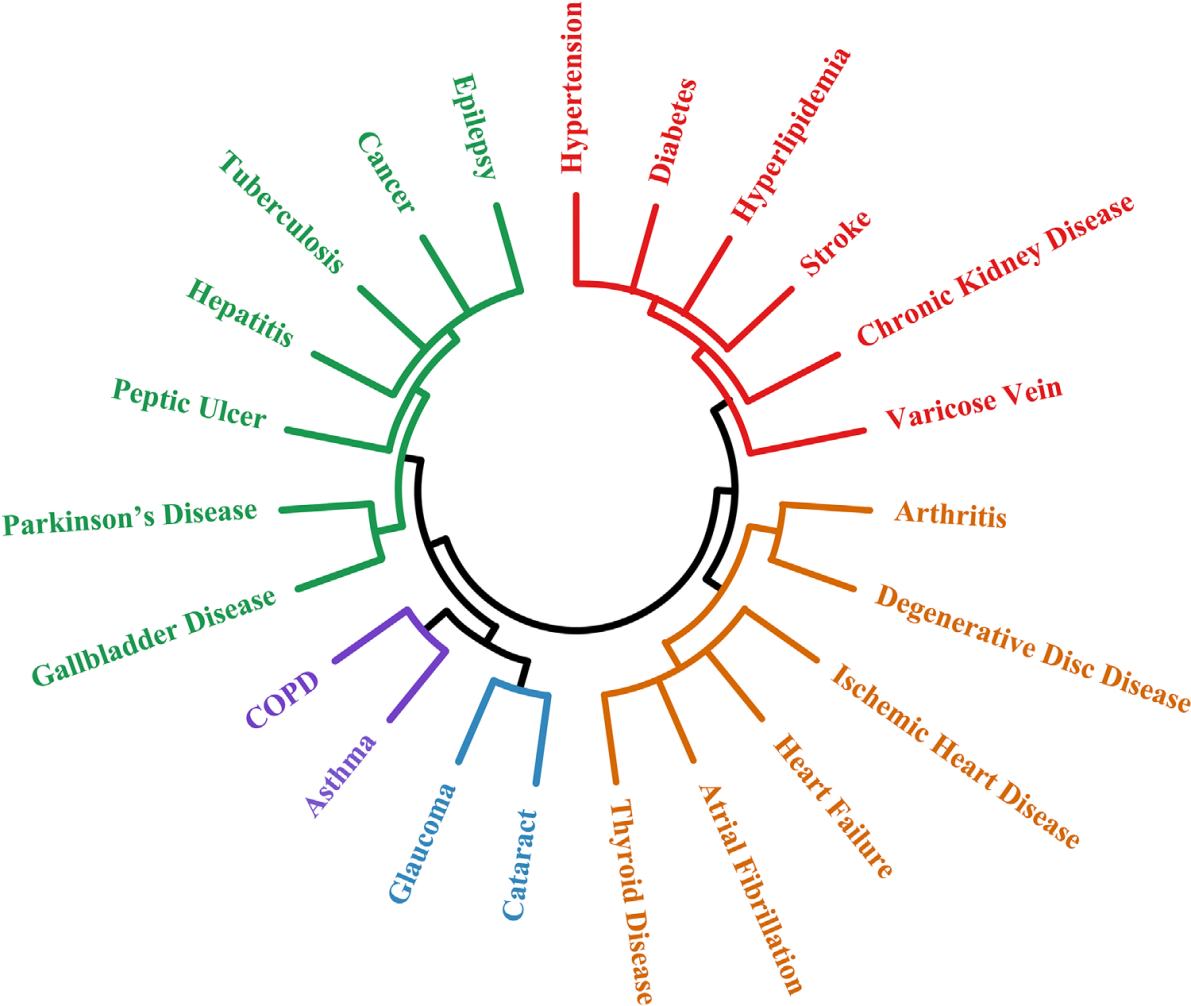
Dendrogram of multimorbidity clusters. Metabolic cluster (red) includes stroke, diabetes, hyperlipidemia, hypertension, chronic kidney disease, and lower extremity varicose veins. Cardiac‐MSK cluster (orange) includes ischemic heart disease, heart failure, atrial fibrillation, thyroid disease, degeneration disc disease, and arthritis. Degenerative ocular cluster (blue) includes cataract and glaucoma. Respiratory cluster (purple) includes chronic obstructive pulmonary disease and asthma. Mixed cluster (green) includes epilepsy, tuberculosis, hepatitis, Parkinson's disease, gall bladder disease, and peptic ulcer.

In total, five hierarchical clusters of multimorbidity were generated from 23 chronic health conditions (Figure 2), that is, the metabolic cluster includes diabetes, hyperlipidemia, hypertension, stroke, chronic kidney disease, and lower extremity varicose veins; the cardiac‐musculoskeletal (MSK) cluster includes ischemic heart disease, heart failure, atrial fibrillation, thyroid disease, degenerative disc disease, and arthritis; the degenerative ocular cluster includes cataract and glaucoma; the respiratory cluster includes chronic obstructive pulmonary disease and asthma; and the mixed cluster includes epilepsy, tuberculosis, hepatitis, Parkinson's disease, gall bladder disease, and peptic ulcer. Of the five multimorbidity clusters, metabolic cluster was significantly associated with an increased likelihood of VaD, but not AD, whereas the degenerative ocular cluster was significantly associated with dementia, and AD in particular, but not VaD. The cardiac‐MSK cluster was associated with VaD. The respiratory cluster and mixed cluster were not significantly associated with any cognitive phenotypes (Table 2).

Persons with MCI were included in the reference groups in the aforementioned analyses. This might affect the estimates of multimorbidity measures in association with dementia and subtypes because MCI represented an intermediate state between normal cognitive aging and dementia. To assess the impact, we repeated the multinomial logistic regression analyses by separating persons with MCI from the reference group, which yielded results with regard to the associations of multimorbidity measures with dementia, AD, and VaD that were overall comparable to those reported in Table 2 (Table S1).

Among dementia‐free participants (*n* = 4968), none of the multimorbidity measures was significantly associated with MCI and aMCI, but the presence of multimorbidity, the greater number of chronic conditions, and the metabolic and cardiac‐MSK clusters were significantly associated with an increased likelihood of naMCI (Table 3).

**TABLE 3:** Associations of multimorbidity and multimorbidity clusters with MCI, amnestic MCI, and non‐amnestic MCI in dementia‐free participants (*n* = 4968).

			MCI		aMCI		naMCI	
Multimorbidity burden and clusters	No. of subjects	No. of people with normal cognition	No. of cases	Odds ratio (95% CI)^a^	No. of cases	Odds ratio (95% CI)^a^	No. of cases	Odds ratio (95% CI)^a^
No. of chronic diseases	4968	3655	1313	1.03 (0.98–1.08)	1106	0.99 (0.94‐1.05)	207	1.25 (1.13‐1.37)^*^, ^†^
Multimorbidity								
No	2008	1509	499	1.00 (reference)	440	1.00 (reference)	59	1.00 (reference)
Yes	2960	2146	814	1.10 (0.96–1.26)	666	1.02 (0.88–1.17)	148	1.68 (1.23–2.30)^*^, ^†^
Multimorbidity clusters^b^								
Metabolic cluster	2800	2030	770	1.10 (0.96–1.26)	629	1.02 (0.88–1.17)	141	1.71 (1.25–2.34)^*^, ^†^
Cardiac‐MSK cluster	1709	1252	457	1.05 (0.90–1.23)	378	0.99 (0.84–1.16)	79	1.54 (1.08–2.18)^*^, ^†^
Degenerative ocular cluster	252	178	74	1.10 (0.81–1.48)	67	1.13 (0.83–1.54)	7	0.88 (0.39–1.98)
Respiratory cluster	363	273	90	0.98 (0.75–1.29)	78	0.95 (0.71–1.26)	12	1.27 (0.67–2.42)
Mixed cluster	438	327	111	1.05 (0.82–1.34)	94	1.00 (0.77–1.30)	17	1.38 (0.79–2.41)

Abbreviations: CI, confidence interval; MCI, mild cognitive impairment; aMCI, amnestic mild cognitive impairment; naMCI, non‐amnestic mild cognitive impairment; MSK, musculoskeletal conditions.

^a^ Odds ratio and 95% confidence interval were derived from logistic regression models that were adjusted for age, sex, education, current smoking, alcohol consumption, and physical inactivity.

^b^ Participants without multimorbidity (n = 2008) were considered the reference group in estimating odds ratios (95% confidence intervals) of mild cognitive impairment and its subtypes associated with various clusters of multimorbidity.

* *p* < 0.05 in uncorrected tests.

^†^ *p* ≤ 0.05 in Bonferroni correction tests for multiple comparison.

After the Bonferroni correction, the associations of the number of chronic diseases with AD, the degenerative ocular cluster with dementia and VaD, and the cardiac‐MSK cluster with VaD became statistically non‐significant (Tables 2 and 3).

### 3.3 Association of multimorbidity with plasma AD‐related biomarkers

An increasing number of chronic diseases was significantly associated with increased plasma Aβ40, Aβ42, and NfL (Table 4). In addition, the presence of multimorbidity was significantly associated with increased plasma Aβ42 and NfL (Table 4). Of the five clusters, the metabolic and degenerative ocular clusters were significantly associated with increased plasma NfL, whereas the cardiac‐MSK cluster was significantly associated with increased plasma Aβ42 (Table 4).

**TABLE 4:** Associations of multimorbidity and multimorbidity clusters with plasma biomarkers (*n* = 1412).

Multimorbidity burden and clusters^a^	No. subjects	β coefficient (95% confidence interval)^b^, plasma biomarkers
Aβ40 (pg/mL)		
No. chronic diseases	1412	0.054 (0.013 to 0.096)^*^, ^[Link]^
Multimorbidity	830	0.056 (−0.050 to 0.163)
Multimorbidity clusters		
Metabolic cluster	791	0.053 (−0.055 to 0.161)
Cardiac‐MSK cluster	468	0.070 (−0.053 to 0.193)
Degenerative ocular cluster	69	0.154 (−0.088 to 0.396)
Respiratory cluster	80	0.059 (−0.171 to 0.288)
Mixed cluster	103	0.137 (−0.067 to 0.341)
Aβ42 (pg/mL)		
No. chronic diseases	1412	0.058 (0.017 to 0.100)^*^, ^[Link]^
Multimorbidity	830	0.108 (0.001 to 0.215)^*^, ^[Link]^
Multimorbidity clusters		
Metabolic cluster	791	0.104 (−0.005 to 0.212)
Cardiac‐MSK cluster	468	0.147 (0.024 to 0.270)^*^, ^[Link]^
Degenerative ocular cluster	69	0.076 (−0.173 to 0.325)
Respiratory cluster	80	0.020 (−0.218 to 0.259)
Mixed cluster	103	0.029 (−0.177 to 0.236)
NfL (pg/mL)		
No. chronic diseases	1412	0.062 (0.023 to 0.101)^*^, ^[Link]^
Multimorbidity	830	0.108 (0.008 to 0.208)^*^, ^[Link]^
Multimorbidity clusters		
Metabolic cluster	791	0.120 (0.018 to 0.221)^*^, ^[Link]^
Cardiac‐MSK cluster	468	0.076 (−0.036 to 0.101)
Degenerative ocular cluster	69	0.333 (0.102 to 0.564)^*^, ^[Link]^
Respiratory cluster	80	0.149 (−0.064 to 0.362)
Mixed cluster	103	−0.109 (−0.295 to 0.077)
Total tau (pg/mL)		
No. chronic diseases	1412	−0.013 (−0.055 to 0.028)
Multimorbidity	830	−0.022 (−0.129 to 0.085)
Multimorbidity clusters		
Metabolic cluster	791	−0.033 (−0.141 to 0.076)
Cardiac‐MSK cluster	468	−0.044 (−0.165 to 0.078)
Degenerative ocular cluster	69	0.131 (−0.117 to 0.379)
Respiratory cluster	80	−0.003 (−0.234 to 0.228)
Mixed cluster	103	0.138 (−0.065 to 0.340)

Abbreviations: Aβ, amyloid‐β; MSK, musculoskeletal conditions; NfL, neurofilament light chain.

^a^ Plasma biomarkers (ie, plasma Aβ40 and NfL) that were not normally distributed were first log transformed using the natural logarithm. Then, all the plasma biomarkers were standardized into z scores.

^b^ β coefficients (95% confidence intervals) were adjusted for age, sex, education, current smoking, alcohol consumption, and physical inactivity.

* *p* ≤ 0.05 in uncorrected tests.

^**^ *p* ≤ 0.05 in Bonferroni tests for multiple comparisons.

^***^ *p* > 0.05 in Bonferroni tests for multiple comparisons.

Following the Bonferroni correction, the association of multimorbidity with Aβ42 and NfL, the metabolic cluster with NfL, and the cardiac‐MSK cluster with Aβ42 became statistically non‐significant (Table 4).

## 4 DISCUSSION

In this population‐based cross‐sectional study of rural‐dwelling Chinese older adults, we found that (1) the presence and burden of multimorbidity were associated with increased likelihoods of dementia, VaD, and naMCI and increased plasma Aβ42 and NfL, while the number of chronic conditions was additionally associated with AD and plasma Aβ40; (2) the metabolic and cardiac‐MSK clusters were associated with VaD and naMCI, whereas the degenerative ocular cluster was associated with AD; and (3) the metabolic, degenerative ocular, and cardiac‐MSK clusters were associated with increased plasma AD‐related biomarkers. Taken together, these results suggest that the burden and certain clusters of multimorbidity are associated with dementia, the main subtypes of dementia, and plasma biomarkers for amyloid and neurodegeneration in older adults.

The association of multimorbidity burden with dementia and MCI was previously reported in population‐based studies,^2^, ^3^, ^5^, ^33^ but the associations of certain multimorbidity clusters with subtypes of dementia and MCI have not yet been explored. We found that, overall, the presence of multimorbidity was associated with VaD and naMCI, but not with AD or aMCI. It is worth noting that multimorbidity as a binary entity was not associated with AD, but the increased number of chronic conditions was associated with both AD and VaD. This was in alignment with the report from the UK Biobank Study,^5^, ^34^ where the load, rather than the presence, of multimorbidity was associated with an increased risk of AD. Furthermore, we had the opportunity to further explore the association of multimorbidity measures with AD‐related plasma biomarkers in a subsample, which showed that the presence of multimorbidity and the number of chronic conditions were associated with increased plasma Aβ42 and NfL, indicating that amyloid and neurodegeneration might partly underline the association of multimorbidity load with dementia.

Defining multimorbidity by clusters could account for disease patterns and diversities.^16^ The population‐based SNAC‐K study in Stockholm, Sweden, found that various patterns of multimorbidity were differentially associated with incident dementia and that certain patterns of multimorbidity (eg, neuropsychiatric, cardiovascular, and sensory impairment/cancer patterns) were linked with an increased risk of dementia.^4^ Data from the UK Biobank Study also reported the associations of different multimorbidity patterns (eg, patterns of cardio‐cerebrovascular/respiratory/metabolic/musculoskeletal/depressive disorders and tumor/genitourinary/digestive disorders) with AD and VaD.^34^ Our study targeted rural older adults in China, and we generated five multimorbidity clusters (ie, metabolic, cardiac‐MSK, degenerative ocular, respiratory, and mixed clusters). Different multimorbidity clusters or patterns were generated in different studies partly due to the fact that different disease spectra could exist across ethnically, culturally, and socioeconomically diverse populations. Notably, our study adds to the current knowledge base by further clarifying the associations of various multimorbidity clusters with MCI and subtypes of MCI.

The close relationships of individual metabolic and cardiovascular diseases with VaD and naMCI have been observed in previous studies.^17^, ^35^, ^36^, ^37^ Of the five multimorbidity clusters, we found that metabolic and cardiac‐MSK clusters were associated with VaD and naMCI, which could be partly due to shared common vascular injury mechanisms such as endothelial dysfunction, inflammation, and vascular remodeling,^35^, ^36^, ^38^ and these mechanisms could also contribute to Alzheimer's pathology and neurodegeneration.^39^, ^40^ In the biomarker subsample, we did find that the metabolic cluster was associated with increased plasma NfL and the cardiac‐MSK cluster was associated with increased plasma Aβ42, which partially supports this pathway.

We found that degenerative ocular cluster was associated with an increased likelihood of AD, but not VaD. This is consistent with the report from the UK Biobank Study, such that participants with cataracts had a higher risk of AD, but not VaD.^41^ The underlying mechanisms are unknown, but evidence has emerged that NfL and Aβ could accumulate in lenses and retinal arterioles of patients with cataracts, MCI, and AD,^42^, ^43^, ^44^ which may partly explain the association between degenerative ocular cluster and AD. In support of this potential pathway, we did find that the degenerative ocular cluster was associated with increased plasma NfL.

Our large‐scale population‐based study engaged rural‐dwelling older adults in China in a low socioeconomic position who received no or very limited formal education, a sociodemographic group that has been substantially underrepresented in epidemiological and clinical research on dementia and AD.^13^ Furthermore, the integration of comprehensive epidemiological, clinical, and cognitive data with AD‐related plasma biomarkers in a subsample allows us to explore the potential neuropathological mechanisms linking multimorbidity load and clusters with cognitive phenotypes. However, our study also has limitations. First, the cross‐sectional nature of the study design does not allow us to infer a causal relationship for any of the observed associations of multimorbidity measures with cognitive phenotypes and plasma biomarkers. Second, we were not able to explore the pathway of tau pathology due to a lack of data on plasma phosphorylated tau proteins (eg, p‐tau181, p‐tau 217, and p‐tau 231). Third, our study involved multiple comparison tests, although driven by predefined hypotheses, which might increase the probability of type I error (ie, false positive and some of the observed associations did not survive the correction of multiple comparisons). Future large‐scale population‐based studies are warranted to replicate this study's findings in different populations. Finally, our study participants were recruited from only one rural region in western Shandong Province. This should be kept in mind when generalizing our research findings to different study populations or settings.

In conclusion, our population‐based study of rural older adults in China suggests that the burden and metabolic and cardiac‐MSK clusters of multimorbidity are associated with increased likelihoods of VaD and non‐aMCI, whereas the degenerative ocular cluster was associated with AD. We further revealed an association of multimorbidity and metabolic and cardiac‐MSK and degenerative ocular clusters with plasma amyloid and NfL. These findings contribute to the current literature with regard to the relationships of multimorbidity burden and clusters with cognitive aging phenotypes (eg, dementia, AD, and MCI) and shed further light on the potential neuropathological mechanisms underlying their associations.

## CONFLICT OF INTEREST STATEMENT

The authors declare no conflicts of interest. Author disclosures are available in the Supporting Information.

## Supporting information

Supplemental Information.

Supplemental Information.

Supplemental Information.

Supplemental Information.
