# Supplementary material for: Multimorbidity, cognitive phenotypes, and Alzheimer's disease plasma biomarkers in older adults: A population‐based study
Source: Alzheimers Dement. 2023 Dec 2;20(3):1550–61. doi: 10.1002/alz.13519 (PMC10984420; doi:10.1002/alz.13519)
Supplement: Supplementary file 2 — Supplemental Information. [file ALZ-20-1550-s002.docx]

**Supplemental Materials**

**Multimorbidity, Cognitive Phenotypes, and Alzheimer’s Disease Plasma Biomarkers in older adults: A Population-based Study**

Yifei Ren^1^, Yuanjing Li^5^, Na Tian^1,2,3,4^, Rui Liu^1^, Yi Dong^1,2,3^, Tingting Hou^1,2,3^, Cuicui Liu^1,2,3^, Xiaolei Han^1,2,3^, Xiaodong Han^1^, Lidan Wang^1^, Davide Liborio Vetrano^5,6^, Tiia Ngandu^7,8^, Alessandra Marengoni^5,8,9^, Miia Kivipelto^8,10,11^, Yongxiang Wang^1,2,3^, Lin Cong^1,2,3*^, Yifeng Du^1,2,3,4*^, Chengxuan Qiu^1,5*^

^1^ Department of Neurology, Shandong Provincial Hospital, Shandong University, Jinan, Shandong 250021, P. R. China.

^2^ Department of Neurology, Shandong Provincial Hospital affiliated to Shandong First Medical University, Jinan, Shandong 250021, P. R. China.

^3^ Shandong Provincial Clinical Research Center for Geriatric Neurological Diseases, Jinan, Shandong 250021, P. R. China.

^4^ Medical Science and Technology Innovation Center, Shandong First Medical University & Shandong Academy of Medical Sciences, Jinan, Shandong 250021, P.R. China.

^5^Aging Research Center and Center for Alzheimer Research, Department of Neurobiology, Care Sciences and Society, Karolinska Institutet-Stockholm University, 171 65 Solna, Sweden.

^6^ Stockholm Gerontology Research Center, 113 46 Stockholm, Sweden

^7^ Department of Public Health and Welfare, Finnish Institute for Health and Welfare, 00271 Helsinki, Finland

^8^ Division of Clinical Geriatrics and Center for Alzheimer Research, Department of Neurobiology, Care Sciences and Society, Karolinska Institutet, 171 77 Solna, Sweden

^9^ Department of Clinical and Experimental Sciences, University of Brescia, 15-25121 Brescia, Italy

^10^ Neuroepidemiology and Ageing Research Unit, School of Public Health, Imperial College London, W2 1PG London, United Kingdom

^11^ Institute of Public Health and Clinical Nutrition, University of Eastern Finland, FI-70210 Kuopio, Finland

**Content**

**Table S1.** Associations of multimorbidity and multimorbidity clusters with mild cognitive impairment, dementia, and subtypes of dementia (n=5,223)

**Figure S1.** The overall and sex-specific prevalence of multiple chronic conditions
