# Supplementary material for: Multimorbidity, cognitive phenotypes, and Alzheimer's disease plasma biomarkers in older adults: A population‐based study
Source: Alzheimers Dement. 2023 Dec 2;20(3):1550–61. doi: 10.1002/alz.13519 (PMC10984420; doi:10.1002/alz.13519)
Supplement: Supplementary file 3 — Supplemental Information. [file ALZ-20-1550-s001.docx]

**Table S1. Associations of multimorbidity and multimorbidity clusters with mild cognitive impairment, dementia, and subtypes of dementia (n=5,223)**

| **Multimorbidity burden and clusters** | **No. of subjects** | **No. of subjects with normal cognition** | **MCI** | |  | **Dementia** | |  | **Alzheimer’s disease** | |  | **Vascular dementia** | |
| --- | --- | --- | --- | --- | --- | --- | --- | --- | --- | --- | --- | --- | --- |
|  |  |  | **No. of cases** | **Odds ratio (95% CI)**^†^ |  | **No. of cases** | **Odds ratio (95% CI)**^†^ |  | **No. of cases** | **Odds ratio (95% CI)**^†^ |  | **No. of cases** | **Odds ratio (95% CI)**^†^ |
| **No. of chronic diseases** | 5,223 | 3,655 | 1,313 | 1.03 (0.98-1.08) |  | 255 | 1.24 (1.13-1.36) |  | 168 | 1.15 (1.03-1.30) |  | 78 | 1.45 (1.27-1.66)^*^ |
| **Multimorbidity** |  |  |  |  |  |  |  |  |  |  |  |  |  |
| **No** | 2,085 | 1,509 | 499 | 1.00 (Reference) |  | 77 | 1.00 (Reference) |  | 57 | 1.00 (Reference) |  | 15 | 1.00 (Reference) |
| **Yes** | 3,138 | 2,146 | 814 | 1.10 (0.96-1.25) |  | 178 | 1.47 (1.10-1.96)^*^ |  | 111 | 1.22 (0.86-1.72) |  | 63 | 2.73 (1.54-4.85)^*^ |
| **Multimorbidity clusters^a^** |  |  |  |  |  |  |  |  |  |  |  |  |  |
| **Metabolic cluster** | 2,971 | 2,030 | 770 | 1.10 (0.96-1.26) |  | 171 | 1.50 (1.12-2.01)^*^ |  | 106 | 1.23 (0.87-1.74) |  | 61 | 2.83 (1.59-5.03)^*^ |
| **Cardiac-MSK cluster** | 1,807 | 1,252 | 457 | 1.05 (0.90-1.23) |  | 98 | 1.32 (0.95-1.84) |  | 69 | 1.25 (0.85-1.83) |  | 27 | 1.91 (0.99-3.67) |
| **Degenerative ocular cluster** | 274 | 178 | 74 | 1.10 (0.81-1.48) |  | 22 | 1.77 (1.03-3.05)^*^ |  | 18 | 1.95 (1.07-3.58)^*^ |  | 4 | 1.77 (0.57-5.56) |
| **Respiratory cluster** | 385 | 273 | 90 | 0.98 (0.75-1.29) |  | 22 | 1.25 (0.73-2.14) |  | 16 | 1.36 (0.72-2.55) |  | 5 | 1.21 (0.42-3.51) |
| **Mixed cluster** | 458 | 327 | 111 | 1.04 (0.82-1.33) |  | 20 | 1.20 (0.81-1.33) |  | 16 | 1.38 (0.75-2.54) |  | 4 | 1.13 (0.37-3.47) |

^†^Odds ratios and 95% confidence intervals were derived from multinomial logistic regression models that were adjusted for age, sex, education, current smoking, alcohol consumption, and physical inactivity, in which individuals with normal cognition (n=3,655) were held as reference category for multinomial logistic modeling.

Abbreviations: MCI, mild cognitive impairment; CI, confidence interval; MSK, musculoskeletal conditions.

^a^Participants without multimorbidity (n = 2,085) were considered the reference group in estimating odds ratios (95% confidence intervals) of cognitive outcomes associated with various clusters of multimorbidity.

^*^p<0.05.
