# Supplementary figures and images for: Multimorbidity, cognitive phenotypes, and Alzheimer's disease plasma biomarkers in older adults: A population‐based study
Source: Alzheimers Dement. 2023 Dec 2;20(3):1550–61. doi: 10.1002/alz.13519 (PMC10984420; doi:10.1002/alz.13519)

**Figure S1. The overall and sex-specific prevalence of multiple chronic conditions**

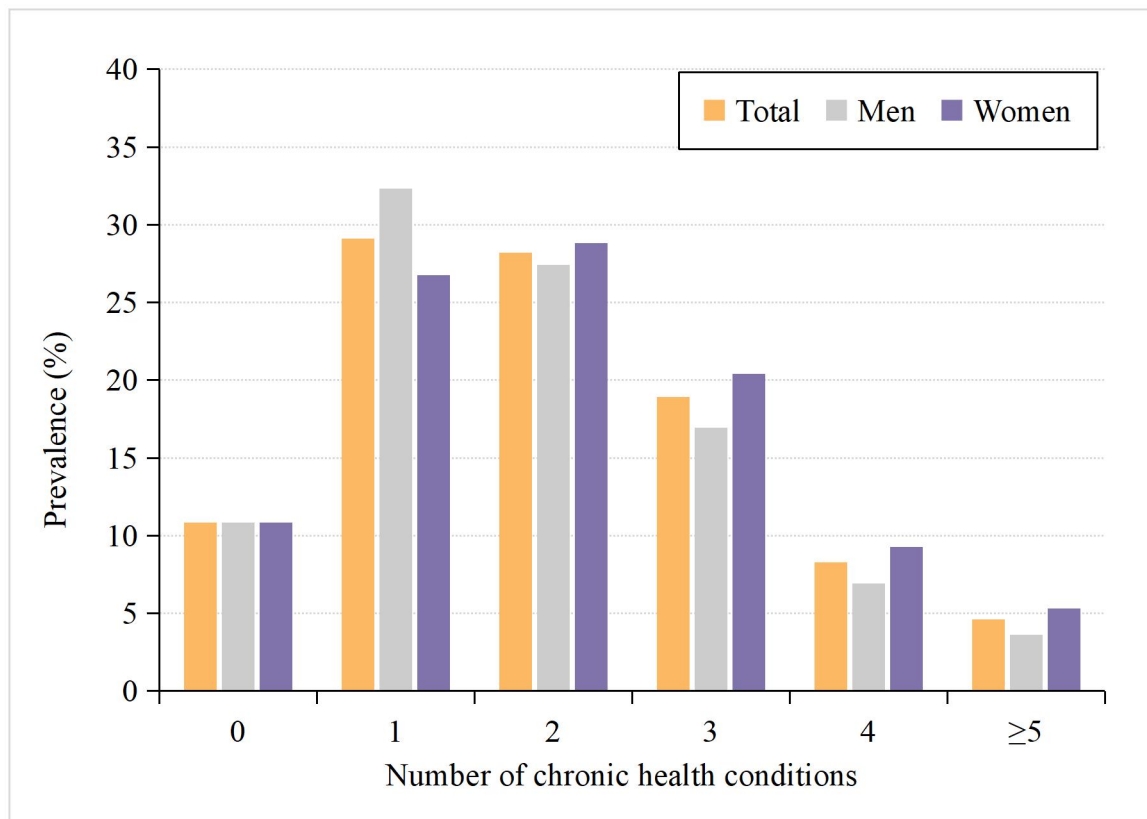

Supplement: Supplementary file 4 — Supplemental Information. [file ALZ-20-1550-s003.pdf]
